# Local recurrence of squamous cell carcinoma of the head and neck after radio(chemo)therapy: Diagnostic performance of FDG-PET/MRI with diffusion-weighted sequences

**DOI:** 10.1007/s00330-017-4999-1

**Published:** 2017-08-15

**Authors:** Minerva Becker, Arthur D. Varoquaux, Christophe Combescure, Olivier Rager, Marc Pusztaszeri, Karim Burkhardt, Bénédicte M. A. Delattre, Pavel Dulguerov, Nicolas Dulguerov, Eirini Katirtzidou, Francesca Caparrotti, Osman Ratib, Habib Zaidi, Christoph D. Becker

**Affiliations:** 1 Department of Imaging, Divisions of Radiology and Nuclear Medicine, Geneva University Hospitals, University of Geneva, Rue, Gabrielle Perret Gentil 4, CH–1211 Geneva 14 Switzerland; 2 Present Address: Department of Radiology, Conception Hospital, Aix-Marseille University, Marseille, France; 3 Centre for Clinical Research, Geneva University Hospitals, Geneva, Switzerland; 4 Division of Clinical Pathology, Geneva University Hospitals, Geneva, Switzerland; 5 Clinic of Otorhinolaryngology Head and Neck Surgery, Geneva University Hospitals, Geneva, Switzerland; 6 Division of Radiation Oncology, Geneva University Hospitals, Geneva, Switzerland

**Keywords:** Head and neck neoplasms, Neoplasm recurrence, local, Imaging, hybrid, Multimodal imaging, Magnetic resonance imaging

## Abstract

**Purpose:**

To determine the diagnostic performance of FDG-PET/MRI with diffusion-weighted imaging (FDG-PET/DWIMRI) for detection and local staging of head and neck squamous cell carcinoma (HNSCC) after radio(chemo)therapy.

**Materials and methods:**

This was a prospective study that included 74 consecutive patients with previous radio(chemo)therapy for HNSCC and in whom tumour recurrence or radiation-induced complications were suspected clinically. The patients underwent hybrid PET/MRI examinations with morphological MRI, DWI and FDG-PET. Experienced readers blinded to clinical/histopathological data evaluated images according to established diagnostic criteria taking into account the complementarity of multiparametric information. The standard of reference was histopathology with whole-organ sections and follow-up ≥24 months. Statistical analysis considered data clustering.

**Results:**

The proof of diagnosis was histology in 46/74 (62.2%) patients and follow-up (mean ± SD = 34 ± 8 months) in 28/74 (37.8%). Thirty-eight patients had 43 HNSCCs and 46 patients (10 with and 36 without tumours) had 62 benign lesions/complications. Sensitivity, specificity, and positive and negative predictive value of PET/DWIMRI were 97.4%, 91.7%, 92.5% and 97.1% per patient, and 93.0%, 93.5%, 90.9%, and 95.1% per lesion, respectively. Agreement between imaging-based and pathological T-stage was excellent (kappa = 0.84, *p* < 0.001).

**Conclusion:**

FDG-PET/DWIMRI yields excellent results for detection and T-classification of HNSCC after radio(chemo)therapy.

***Key points*:**

• *FDG-PET/DWIMRI yields excellent results for the detection of post-radio(chemo)therapy HNSCC recurrence.*

• *Prospective one-centre study showed excellent agreement between imaging-based and pathological T-stage.*

• *97.5% of positive concordant MRI, DWI and FDG-PET results correspond to recurrence.*

• *87% of discordant MRI, DWI and FDG-PET results correspond to benign lesions.*

• *Multiparametric FDG-PET/DWIMRI facilitates planning of salvage surgery in the irradiated neck.*

## Introduction

Patients with squamous cell carcinoma of the head and neck (HNSCC) are treated with radio(chemo)therapy, surgery or with a combination thereof [1, 2]. It has been suggested that up to 50% of patients with HNSCC will experience disease relapse during their lifetime, locoregional recurrence being more common than distant metastases or second primary tumours. Local recurrence constitutes an important prognostic factor and influences the 5-year survival rate and quality of life [2, 3]. Early diagnosis of local recurrence and precise depiction of tumour extent are important since surgical salvage increases overall survival [4].

Locally recurrent HNSCC is often more difficult to detect than primary SCC. Endoscopy may fail in the presence of submucosal recurrence and findings at cross-sectional imaging may be confusing since radio(chemo)therapy may induce morphological, functional and metabolic changes that are difficult to interpret [1, 5, 6]. Nonetheless, previous studies have suggested that both magnetic resonance imaging (MRI) with diffusion-weighted imaging (DWI) sequences and FDG positron emission tomography computed tomography (PET/CT) can substantially improve the detection of recurrent HNSCC [5–9]. As the combined use of PET/CT and MRI with DWI can add diagnostic certainty in difficult post-treatment situations [1, 9], hybrid PET/MRI systems have raised high hopes in the field of oncological imaging due to the potential to obtain morphological, functional and metabolic information in a single examination [10–20]. Previous studies have addressed important technical questions related to PET/MRI feasibility in the head and neck (HN), quantification of FDG uptake with PET/MRI as compared to PET/CT and workflow issues [12, 13, 16–19]. Since hybrid PET/MRI systems are expensive and examination protocols may involve long scanning times, the usefulness of PET/MRI in clinical routine needs to be determined based on added clinical value [20]. However, the added clinical value of hybrid PET/MRI examinations awaits validation in several oncological applications, including HN tumours [10, 15].

The purpose of this prospective study was to assess the diagnostic performance of FDG-PETMRI with DWI for the detection and T-classification of HNSCC recurrence in a series of consecutive patients treated with radio(chemo)therapy.

## Materials and methods

### Patient population

This prospective clinical study was approved by the institutional ethics committee and performed in accordance with the guidelines of the Helsinki II declaration. Written informed consent was obtained from all subjects. Eligible patients were identified at the Clinic for Otorhinolaryngology, Head and Neck Surgery of the University Hospital of Geneva. Over 36 months, hybrid PET/MRI examinations were performed in a consecutive series of 76 adult patients previously treated with curative radio(chemo)therapy ± surgery (delay between treatment end and imaging: mean ± SD = 15.2 ± 12.8 months, median [quartiles] = 12 months [5–22.5]). Indications for PET/MRI were persisting or newly developed symptoms after radio(chemo)therapy (pain, reflex otalgia, hoarseness, dysphagia). Exclusion criteria were standard MRI contraindications. None of the potentially eligible patients refused to participate. Two PET/MRI examinations were excluded from the study due to poor image quality (n = 1) or absent follow-up (n = 1). Therefore, a total of 74 PET/MRI examinations formed the basis of this series. Most patients (50/74, 67.5%) were males (mean age ± SD 62.1 ± 12.6 years). A small proportion of this cohort (15/74 patients) was included in a study comparing image quality and whole-body FDG uptake detectability with PET/MRI versus PET/CT [16].

### Image acquisition

PET/MRI examinations were performed on a Philips Ingenuity time of flight (TF) hybrid PET/MRI (Philips Healthcare, Cleveland, OH, USA) [12]. All patients fasted >4 h prior to injection of 3.5 MBq/kg body weight FDG. The time interval necessary for FDG uptake was used for HN MRI scanning including a sequence for PET attenuation correction (AC). HN MRI obtained with a 16-channel SENSE neurovascular coil covered the area from the roof of the frontal sinuses to the aortic arch. The following high-resolution sequences were acquired: coronal STIR (TR/TE/TI = 5,043/80/200 ms; voxel = 0.45 × 0.45 × 4 mm^3^, 3 min 30 s), axial T2 (TR/TE = 3,528/90 ms; voxel = 0.45 × 0.45 × 3 mm^3^, 2 min 40 s), axial SE EPI-DWI with six diffusion gradient b values (TR/TE/TI = 6,803/72/230 ms, b = 0, 50, 100, 500, 750, 1,000; voxel = 1.3 × 1.3 × 3 mm^3^, 4 min 05 s) and with apparent diffusion coefficient (ADC) map calculation by mono-exponential fitting [1, 5–7], axial and coronal T1(TR/TE = 683/16 ms; voxel = 0.45 × 0.45 × 3 mm^3^, 3 min 45 s) before and after injection of gadoterate-meglumine (0.1 mmol/kg Dotarem, Guerbet, Aulnay-sous-Bois, France), and contrast-enhanced axial 3DT1GE Dixon(flip angle 10°, TE_1_/TE_2_//TR = 1.44/2.6/5.7 ms, voxel = 0.45 × 0.45 × 1.5 mm^3^, 4 min 12 s). We used a 6b-value SE EPI-DWI sequence because a similar 6b-value SE EPI-DWI sequence has been successfully used by other authors [7, 21–23]. All commercially available DWI sequences use fat saturation, which can be obtained by chemical shift selective fat saturation, water excitation or by STIR methods. Based on the literature [24, 25] and on our experience, DWI with STIR-based fat saturation is more robust in the HN than classical spectral fat saturation and yields good quality images. After HN MRI, a whole-body 3DT1GE Dixon (flip angle 10°, TE_1_/TE_2_//TR = 1.12/2.1/3.3 ms, voxel = 0.78 × 0.78 × 6 mm^3^, 19 s/stack, 8–10 stacks) and an AC sequence (2 min 30 s) were acquired. Whole-body PET acquisition was started 60 min post-injection (10 beds, acquisition = 32 min). PET images were corrected for attenuation using the segmented MRI-based AC procedure described in the literature [26]. PET reconstruction was performed using a 3D-LOR-TF-blob-based OSEM algorithm (3 iterations, 33 subsets, voxel = 2 × 2 × 2 mm^3^ for HN).

### Image evaluation, diagnostic criteria and measurements

Two board-certified radiologists with substantial experience in HN MRI and PET/CT (>15 years) and a board-certified nuclear medicine physician with substantial experience in PET/CT and HN MRI (>10 years) evaluated the images separately and were blinded to all clinical/histopathological data. In case of discrepant evaluations, consensus was reached. Findings were recorded on pre-defined evaluation sheets using a five-point scale for receiver operating characteristics (ROC) analysis as follows: 1, definitely negative for recurrence; 2, probably negative; 3, indeterminate, therefore, suspicious/possibly positive; 4, probably positive; and 5, definitely positive.

The three readers evaluated morphological MRI first, then DWI and PET. All images (MRI, DWI and PET) were assessed according to the diagnostic criteria established in the literature and taking into consideration diagnostic pitfalls related to radiation-induced changes [1, 9]. Internationally established qualitative and quantitative criteria were applied [1, 5–9, 11, 27, 28]. Tumours involving the upper aero-digestive tract, the neopharynx (after total laryngectomy) or flaps in the oral cavity/pharynx were considered as local recurrence [27]. On MRI, recurrent tumours were diagnosed in the presence of well-defined or ill-defined mass-like lesions with intermediate T2 signal (‘evil grey’), moderate contrast enhancement and restricted diffusivity (high signal on b1000, low signal on ADC) [1, 5–7, 27, 28]. Lesions with high signal on T2, strong contrast enhancement and high signal on b1000 and ADC were interpreted as suggesting post-treatment inflammatory oedema. Mature scar tissue/long-standing fibrosis was diagnosed in the presence of an elongated lesion with very low signal on T2, no/minor contrast enhancement, and low signal on b1000 and ADC [1]. If on a DWI sequence localised artefacts were seen on slices outside the lesion to be measured, the sequence was regarded as being of acceptable quality and ADC measurements were carried out. Qualitative DWI assessment (visual assessment of b1000 and ADC) and quantitative assessment with ADC threshold were obtained for all lesions. The ADC threshold was calculated after completed radiological-histological correlation based on ROC analysis of prospectively measured ADCs [22]. Focal FDG uptake (visual tracer accumulation exceeding the adjacent background activity) was rated as PET positive taking into account physiological FDG accumulation and pitfalls in the HN, such as muscular, salivary gland, physiological Waldeyer’s ring uptake or post-treatment inflammatory changes [1, 8, 9, 20, 29–31]. Qualitative and quantitative PET assessment (with standardised uptake value (SUV) threshold) was obtained. The SUV threshold was calculated analogous to the ADC threshold.

Benign post-treatment lesions and complications (oedema, scar/fibrosis, soft tissue- and osteonecrosis, ulceration, denervation atrophy) were diagnosed on combined PET/DWIMRI taking into consideration established criteria [1]. As FDG uptake can be variable in post-radiotherapy changes/complications, increased focal FDG uptake was not necessarily regarded as indicating recurrence, and MRI and DWI characteristics were taken into consideration for the combined PET/DWIMRI interpretation [1].

Measurements were obtained for: diameters for tumours and benign lesions, mean/minimum ADC values (ADCmean/ADCmin), and mean/maximum standardised uptake values (SUVmean/SUVmax). Tumour ADCs were measured with small elliptical regions of interest (ROIs) placed over several tumour sections on b1000 images and copied on the corresponding ADCmaps, while carefully avoiding areas of apparent necrosis [1, 27, 31]. Average ADCmean/ADCmin values were then calculated for each measured tumour. In analogy, SUV measurements were performed with ROIs placed on anatomically matched areas [16, 31].

### Standard of reference and correlation with imaging findings

The standard of reference consisted of histology and follow-up ≥24 months after PET/DWIMRI. Histology was obtained within 2 weeks: (1) in lesions with a rating ≥3 on PET/DWIMRI, (2) in endoscopically suspicious lesions or (3) whenever there was a discrepancy between clinical/endoscopic examination and imaging. Histology included endoscopic biopsy and salvage surgery. Histological analysis of the resected tumours was based on serial whole-organ sections as described in the literature [28]. It served as a gold standard for the assessment of the pathological T-stage (pT) according to UICC [32]. Two experienced pathologists (>12 years) interpreted histology prospectively and blinded to imaging findings.

Patients with negative examinations or negative histology were followed ≥24 months to determine whether negative readings corresponded to true negative assessments and to detect false-negative evaluations. Follow-up consisted of clinical evaluation and fiberoptic endoscopy every month during the first year, every 2 months in the second year, every 3 months in the third year, every 6 months in the fourth year and additional cross-sectional imaging. If follow-up was negative during the entire period, negative assessments were considered as true negatives. If recurrence was proven ≤3 months after PET/MRI, negative assessments were considered as false negatives. If recurrence was proven >3 months after a negative PET/MRI, the case was re-evaluated at the interdisciplinary HN tumour board to distinguish between a false-negative evaluation and a metachronous tumour unrelated to the initial PET/DWIMRI.

After completed image analysis, correlation between follow-up, histopathological and imaging findings was obtained. Correlation between imaging and whole-organ surgical specimens was made on a slice-by-slice basis.

### Statistical analysis

Statistical analysis was carried out by an experienced biomedical statistician (>15 years). Diameters, ADCmean/ADCmin and SUVmean/SUVmax for benign lesions and tumour recurrence were compared using a linear mixed effect regression model with a random intercept to account for data clustering. The diagnostic performance for combined PET/DWIMRI was assessed globally by calculating the area under the curve (AUC) and specifically at a cut-off of 3 (sensitivity, specificity, predictive values, accuracy). Statistical comparisons considered paired clustered data [33, 34]. An optimal cut-off value for ADCmean/SUVmean was calculated by minimising the distance between the corresponding point of the ROC curve and the upper left graph corner [35]. Multivariant logistic regression analysis (with mixed effects to account for clustering) was performed to assess the association between histology and ADCmean/SUVmean binarised according to optimal cut-off values. Cohen’s kappa coefficient was used to assess the concordance between PET/DWIMRI and the pathological T-classification (pT) [36]. All statistical analyses were conducted with R3.3.1(R-foundation for Statistical Computing, Vienna, Austria) and statistical tests were two-sided with a significance level of 0.05.

## Results

### Descriptive statistics for local recurrence and benign post-treatment lesions

**Fig. 1:**
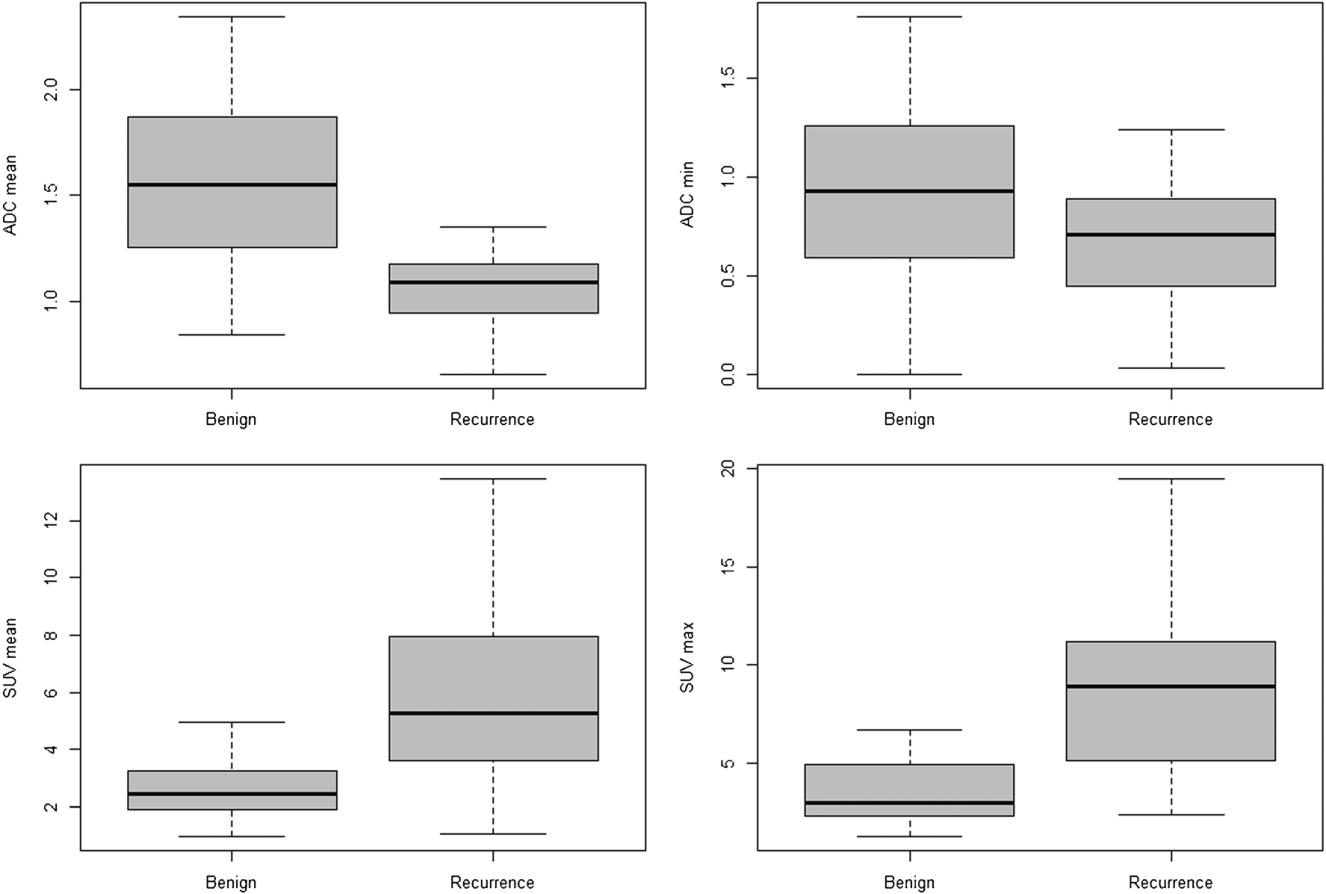
Box plots for ADCmean and ADCmin (in 10^-3^ mm^2^/s), SUVmean and SUVmax values for local tumour recurrence and benign post-treatment lesions. Median values (black lines), first and third quartiles, and whiskers indicating data minimum and maximum. Median values for tumour recurrence (interquartile range): ADCmean = 1.09 (0.95–1.18), ADCmin = 0.71 (0.45–0.89), SUVmean = 5.27 (3.62–7.93), SUVmax = 9.29 (5.03–11.04). Median values for benign lesions (interquartile range): ADCmean = 1.55 (1.25–1.87), ADCmin = 0.93 (0.59–1.26), SUVmean = 2.47 (1.91–3.25), SUVmax = 3.57 (2.52–5.3). The difference between median values for recurrent tumours and benign post-treatment lesions was statistically significant (*p* < 0.001)

There were no adverse effects from performing PET/DWIMRI or the standard of reference. All images, including DWI, were considered of good/acceptable quality for interpretation. Histology and additional follow-up were the proof of diagnosis in 46/74 (62.2%) patients while follow-up alone (mean ± SD = 34 ± 8 months) was the proof of diagnosis in 28/74 (37.8%). Based on the standard of reference, 43 locally recurrent tumours (37 salvage surgery, six endoscopic/excisional biopsy) were present in 38 patients (one SCC in 34 patients; two SCCs in three patients; three SCCs in one patient), while 36 patients had only benign post-treatment lesions/complications. According to UICC [32], recurrent tumours were classified as pT4 (n = 22), pT3 (n = 8), pT2 (n = 3), pT1 (n = 9) and pTis (n = 1). Histological differentiation was as follows: well-differentiated (n = 8; 18.6%), moderately differentiated (n = 27; 62.8%), poorly differentiated (n = 7; 16.3%) and not assessable (one *in situ* tumour). Sixty-two benign lesions in 46 patients (36 patients without and ten with recurrence) included: mucositis/inflammation (n = 35), infection/abscess (n = 3), fibrosis/scars (n = 11), soft tissue necrosis/fistula/granulation tissue (n = 6), muscle denervation (n = 2), osteoradionecrosis (n = 3) and parakeratosis (n = 2). Descriptive statistics for local recurrence and benign lesions are shown in Table 1 and Fig. 1.

**Table 1:** Descriptive statistics for local tumour recurrence and benign post-treatment lesions

	All	Benign lesions	Local tumour recurrence
Lesions (N)	105	62	43
Lesion localisation, n/N (%)			
Oral cavity	29/105 (27.6%)	16/62 (25.8%)	13/43 (30.2%)
Oropharynx	25/105 (23.8%)	16/62 (25.8%)	9/43 (21%)
Larynx	24/105 (22.8%)	11/62 (17.8%)	13/43 (30.2%)
Hypopharynx	11/105 (10.5%)	7/62 (11.3%)	4/43 (9.3%)
Nasopharynx	11/105 (10.5%)	10/62 (16.1%)	1/43 (2.3%)
Paranasal sinuses	5/105 (4.8%)	2/62 (3.2%)	3/43 (7%)
Lesion diameters, mm (mean ± SD)			
Long transverse diameter*		20.8 ± 11.1	27.5 ± 14.4
Short transverse diameter*		11.4 ± 7.3	15.4 ± 10.2
Cranio-caudal diameter		19.6 ± 15.1	23.5 ± 11.7
ADCmean (×10^-3^ mm^2^/s)			
mean ± SD*		1.59 ± 0.42	1.10 ± 0.24
ADCmin (×10^-3^ mm^2^/s)			
mean ± SD*		0.93 ± 0.43	0.66 ± 0.30
SUVmean			
mean ± SD*		2.91 ± 1.49	5.91 ± 2.87
SUVmax			
mean ± SD*		4.09 ± 2.83	8.79 ± 4.75

*Statistically significant difference between benign lesions and local tumour recurrence

Comparisons between benign post-treatment lesions and tumour recurrence for: ADCmean: *p* < 0.0001; ADCmin: *p* = 0.0004 ; SUVmean: *p* < 0.0001 ; SUVmax: *p* < 0.0001 ; longitudinal transverse diameter : *p* = 0.0097; short transverse diameter : p = 0.0072; cranio-caudal diameter: p = 0.1450

Indicated p values for statistical comparison take the effect of clustering into consideration

*ADC* apparent diffusion coefficient; *SUV* standardised uptake value

### Diagnostic performance of PET/DWIMRI

**Fig. 2:**
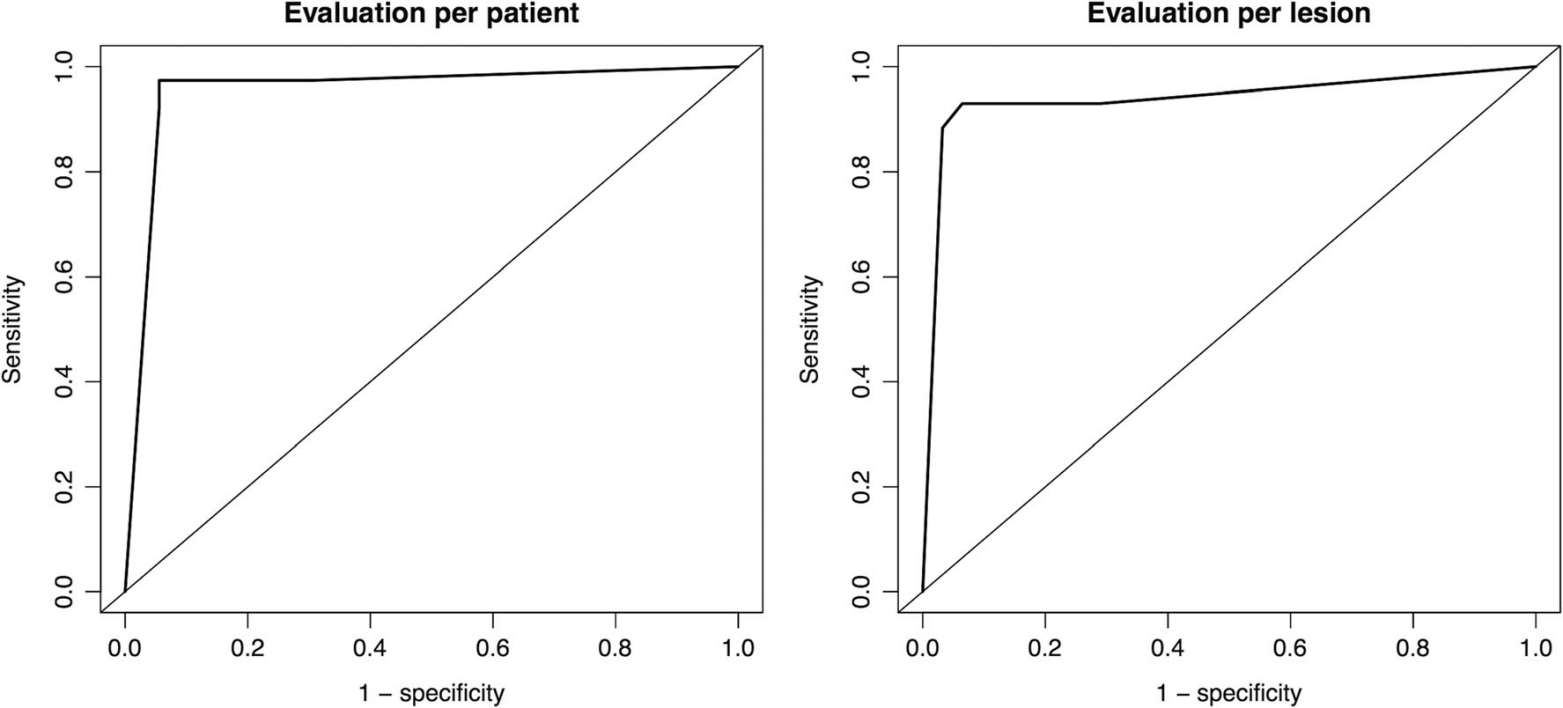
ROC curves for combined PET/DWIMRI for the detection of local HNSCC recurrence after radio(chemo)therapy. Patient-per-patient evaluation: AUC (95% CI) PET/DWIMRI = 0.954 (0.903–1.000). Lesion-per-lesion evaluation: AUC (95% CI) PET/DWIMRI = 0.939 (0.887–0.990). The ROC curve for lesions was calculated taking data clustering into consideration

The AUC for PET/DWIMRI was 0.954 (95% confidence interval (CI) 0.903–1.000) in the patient-per-patient evaluation and 0.939 (0.887–0.990) in the lesion-per-lesion evaluation, respectively (Fig. 2). The diagnostic performance of PET/DWIMRI for a cut-off of3 is summarised in Table 2.

**Table 2:** Diagnostic performance of combined positron emission tomography (PET)/diffusion-weighted imaging (DWI) MRI for the detection of head and neck squamous cell carcinoma recurrence for a cut off of 3 in the patient-by-patient analysis (N = 74) and in the lesion-by-lesion analysis (N = 105)

	Combined PET/DWIMRI patient-by-patient analysis	Combined PET/DWIMRI lesion-by-lesion analysis
Total number of patients/lesions	74	105
True positive, n	37	40
True negative, n	33	58
False positive, n	3	4
False negative, n	1	3
Sensitivity, % (95% CI)	37/38, 97.4% (86.2–99.9)	40/43, 93.0% (85.4–100.0)
Specificity, % (95% CI)	33/36, 91.7% (77.5–98.2)	58/62, 93.5% (87.5–99.6)
Positive predictive value, % (95% CI)	37/40, 92.5% (79.6–98.4)	40/44, 90.9% (82.6–99.2)
Negative predictive value, % (95% CI)	33/34, 97.1% (84.7–99.9)	58/61, 95.1% (90.1–100.0)
Accuracy, % (95% CI)	70/74, 94.6% (86.7–98.5)	98/105, 93.3% (89.0–97.7)

Results of prospective assessments (without ADC or SUV thresholds) are shown. Results are reported with percentages (%) and 95% confidence intervals (95% CI)

*ADC* apparent diffusion coefficient, *SUV* standardised uptake value

### Analysis of ADC and SUV values

**Fig. 3:**
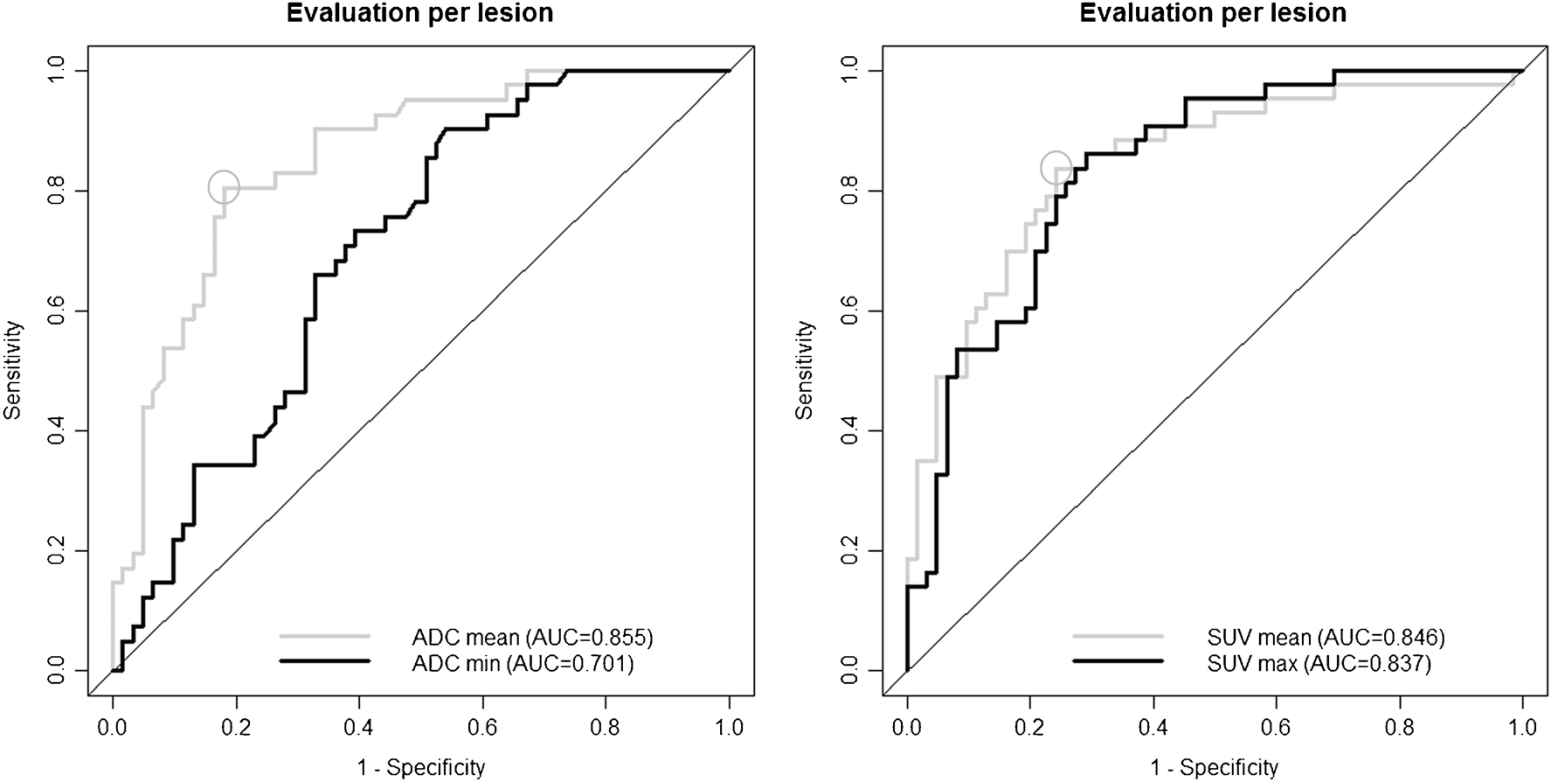
ROC curves for ADC and SUV values for the detection of local recurrence. ADC and corresponding SUV values were measured in 105 lesions. AUC (95% CI) ADCmean = 0.855 (0.785–0.924), AUC (95% CI) ADCmin = 0.701 (0.606–0.796), AUC (95% CI) SUVmean = 0.846 (0.775–0.917), AUC (95% CI) SUVmax = 0.837 (0.766–0.909). There was no statistically significant difference between AUCs for SUVmean versus SUVmax (*p* = 0.763), ADCmean versus SUVmean (*p* = 0.7696) and ADCmean versus SUVmax (*p* = 0.6067). However, comparison between ADCmean versus ADCmin, revealed a statistically significant difference (*p* = 0.0005). All above-mentioned AUCs were inferior to the AUC of combined PET/DWIMRI (*p* < 0.01). The optimal cut-off values for ADCmean and SUVmean are indicated by circles

The ROC curves for ADCmean/ADCmax and SUVmean/SUVmax values for the detection of local recurrence are shown in Fig. 3. The respective AUCs were inferior to the AUC of combined PET/DWIMRI (*p* < 0.01). The optimal cut-off value for ADCmean and SUVmean was 1.208 × 10^-3^ mm^2^/s and 3.361, respectively (Fig. 3). For ADCmean ≤1.208, the sensitivity for the detection of recurrence was 80.5% (70.2–90.8) and the specificity was 82.0% (70.7–93.2). For SUVmean ≥3.361, the sensitivity was 83.7% (72.3–95.1) and the specificity was 75.8% (62.5–89.1), respectively. For the combination ADCmean ≤1.208 *and* SUVmean ≥3.361 (combination is positive if *both* criteria are positive), the sensitivity for the detection of recurrence was 63.8% (54.2–82.4) and the specificity was 75.8% (87.7–99.2). For the combination ADCmean ≤1.208 *or* SUVmean ≥3.361 (combination is positive if *at least one* criteria is positive), the sensitivity was 95.1% (88.5–100.0) and the specificity was 63.9% (48.3–79.5). Multivariate regression analysis showed that ADCmean ≤1.208 and SUVmean ≥3.361 were independently associated with histology with odds ratios equal to 13.62 (4.08–45.42) (p < 0.001) and 11.10 (2.58–47.71) (p = 0.001), respectively. Therefore, each binarised criterion independently and significantly improved the detection of recurrence when added to the other criterion.

Comparison of AUCs of PET/DWIMRI with ADCmean/SUVmean threshold versus PET/DWIMRI without threshold (qualitative assessment) revealed no statistically significant difference (*p* > 0.05). Comparisons took data clustering into consideration.

SUVmean and ADCmean values were not correlated (Spearman’s correlation coefficient rho = 0.12, *p* = 0.4468).

### Correlation between imaging findings and the standard of reference

**Fig. 4:**
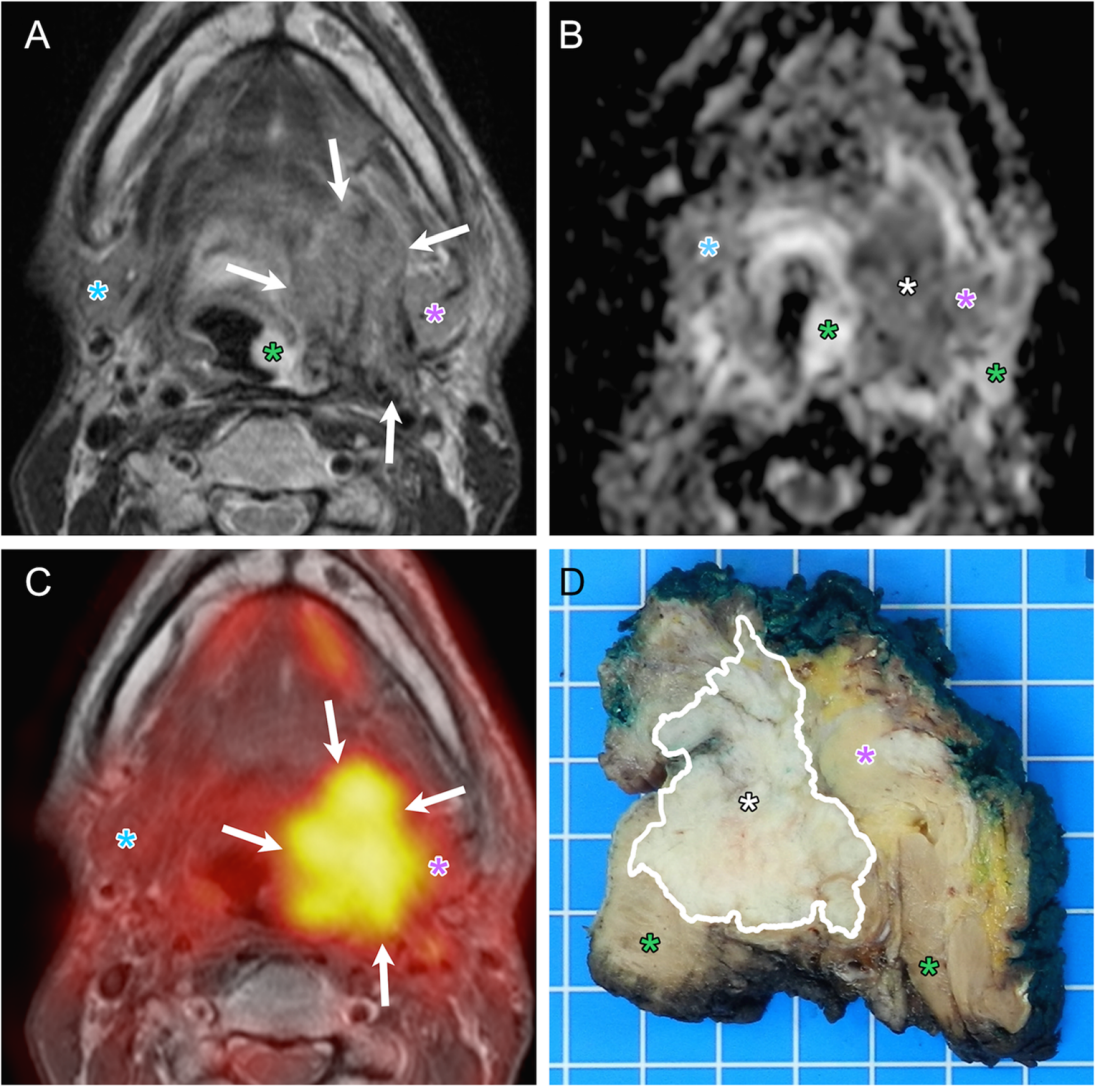
True positive evaluation with combined PET/DWIMRI (positive concordant findings on MRI, DWI and PET). A 48-year-old male with reflex otalgia 1 year after radiochemotherapy for squamous cell carcinoma of the base of the tongue. Endoscopy: oedema and intact mucosa. T2 (**A**): infiltrative tumour recurrence with intermediate signal (arrows) in the left tongue base, extrinsic tongue muscles, vallecula and parapharyngeal space. Suspected invasion of the left submandibular gland (pink asterisk). Submucosal oedema with very high T2 signal (green asterisk). Normal right submandibular gland (blue asterisk). ADC map (**B**): restricted diffusion suggesting recurrence (white asterisk, ADCmean = 1.127 × 10^-3^ mm^2^/s). High ADC signal surrounding the tumour (green asterisks, ADCmean = 1.789–1.965 × 10^-3^ mm^2^/s) due to oedema. Left and right submandibular glands (pink and blue asterisks). (**C**) PET/MRI (PET fused with T1) suggests recurrence (increased FDG uptake, arrows, SUVmean = 7.688; SUVmax = 12.11). (**D**) Whole-organ axial section from surgical specimen (same orientation) confirms recurrence (white asterisk) invading the above-described structures. Submandibular gland (pink asterisk). Tumour margins contoured by pathologist (white line). Green asterisks: inflammatory oedema. T-stage on PET/DWIMRI was T4a. Pathological stage was pT4a

**Fig. 5:**
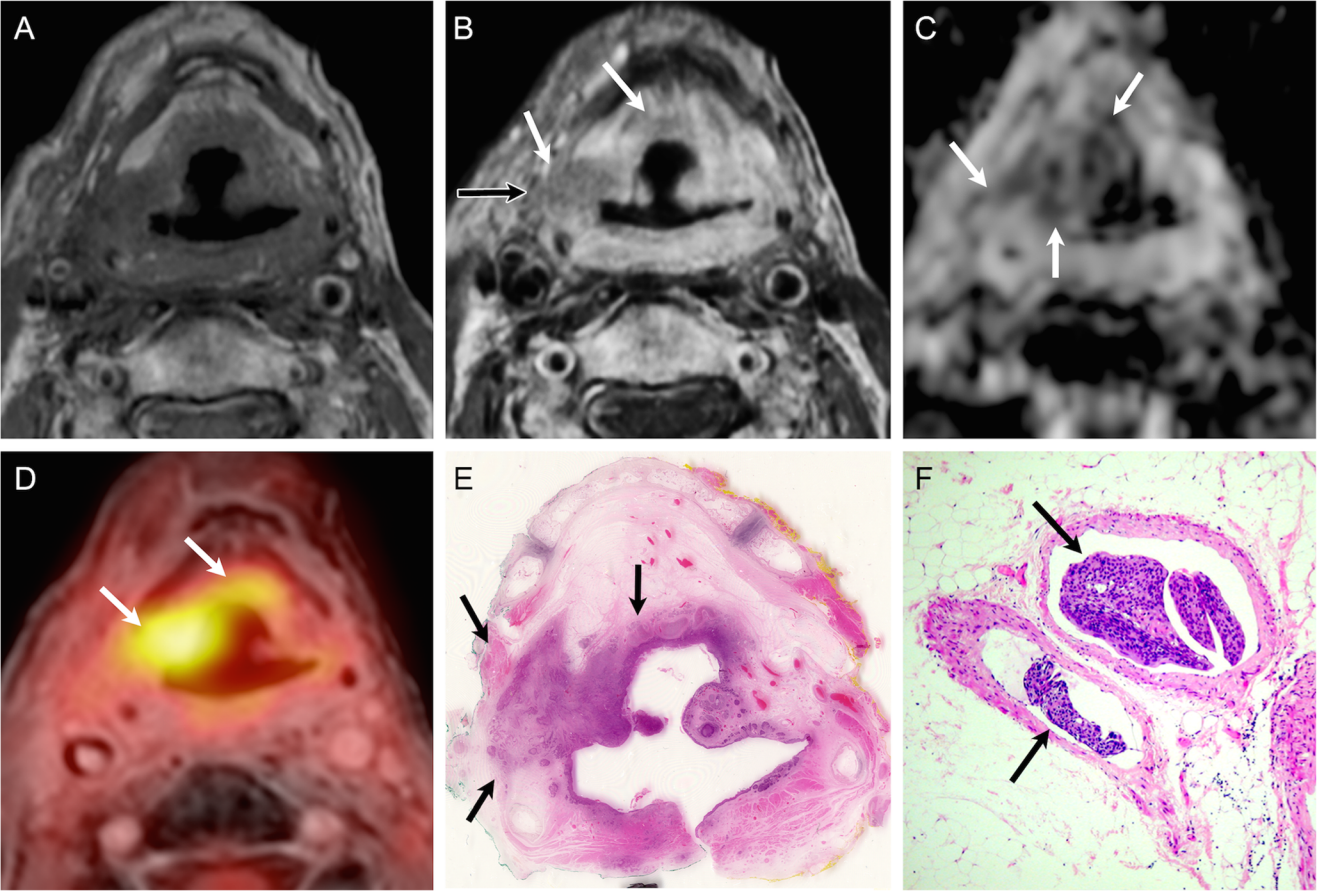
True positive evaluation with combined PET/DWIMRI (positive concordant findings on MRI, DWI and PET). Sixty-nine-year old male with pain 4 years after radiochemotherapy for SCC of the hypopharynx. Unenhanced T1 (**A**): poorly defined hypointensity in both aryepiglottic folds, pre-epiglottic space and retropharyngeal space. Contrast-enhanced T1 (**B**): infiltrative, moderately enhancing lesion (white arrows) in the right paraglottic and pre-epiglottic space with invasion into the soft tissues of the neck (black arrow) suggesting recurrence. Note strongly enhancing retropharygeal space and left aryepiglottic fold due to inflammatory oedema. (**C**) ADC map: restricted diffusion on the right (arrows, ADCmean = 0.997–10^-3^ mm^2^/s) consistent with recurrence. High signal in the left paraglottic space and retropharyngeal space (ADCmean = 1.815 × 10^-3^ mm^2^/s) due to inflammatory oedema. (**D**) PET/MRI (PET fused with gadolinium-enhanced Dixon) consistent with recurrence (arrows, SUVmean = 4.417; SUVmax = 5.518). (**E**) Corresponding whole-organ axial histological section (haematoxylin-eosin, HE) confirms recurrence on the right (arrows) and inflammatory oedema on the left and in the retropharyngeal space. (**F**) Section from right specimen periphery (HE, original magnification 100×) depicts venous tumour thrombi. T stage on PET/DWIMRI was T4a. Pathological stage was pT4a

**Fig. 6:**
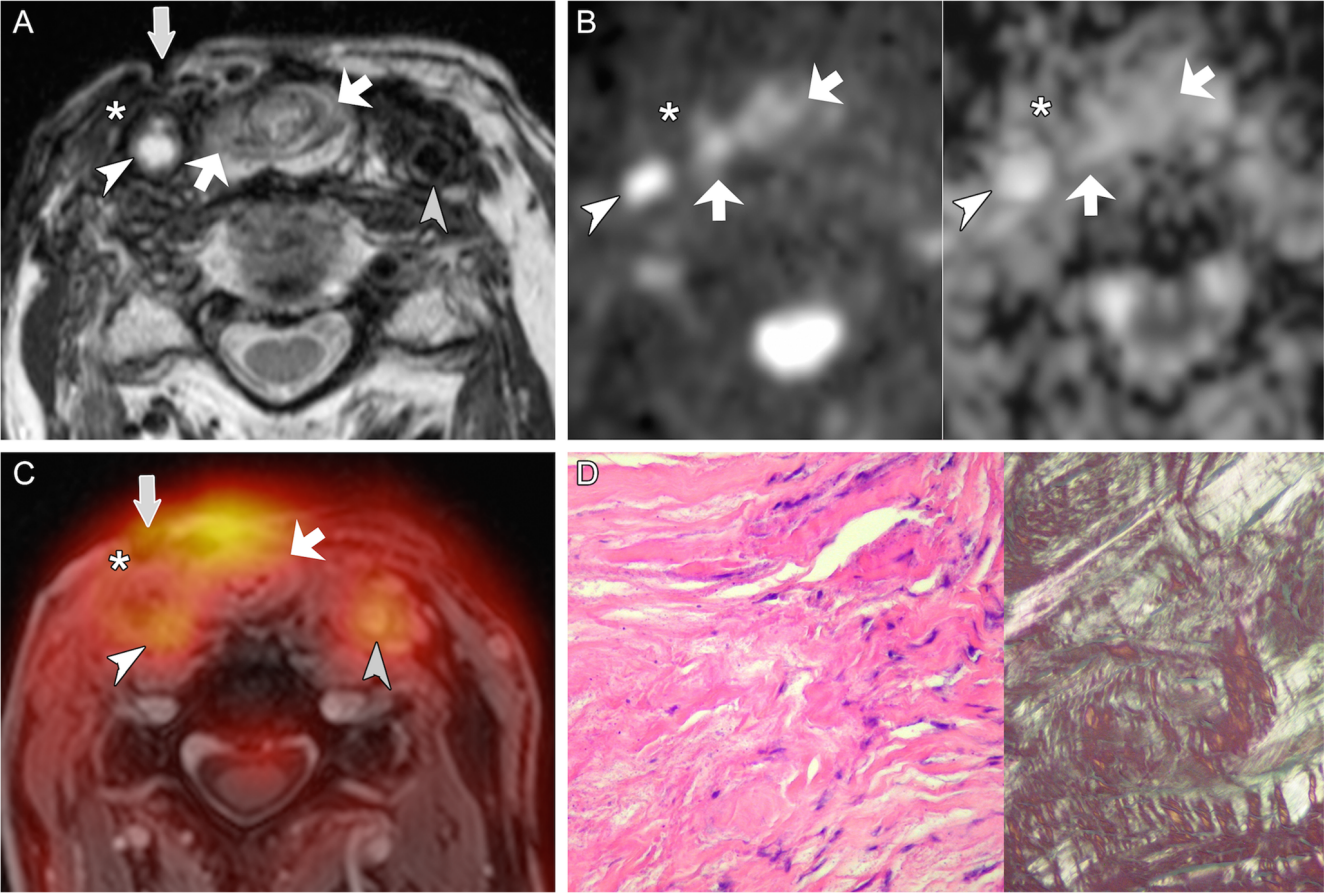
True negative evaluation with combined PET/DWIMRI (discordant findings on MRI, DWI and PET). A 77-year-old male with pain and neck fistula 5 years after total laryngectomy and radiotherapy for squamous cell carcinoma of the larynx. T2 (**A**): neopharynx with intermediate signal (oblique arrows) suggesting probable recurrence and fistula connecting the neopharynx to the skin (vertical arrow). Surrounding long-standing fibrosis with strong T2 hypointensity (asterisk). White arrowhead: occluded common carotid artery (CCA). Grey arrowhead: left normal CCA. (**B**) DWI with b1000 (left) and ADC map (right): absent restriction of diffusion in the neopharynx (arrows) and along the fistulous tract (ADCmean = 1.578–1.692 × 10^-3^ mm^2^/s). Area of fibrosis (asterisk). Occluded right CCA (arrowhead). (**C**) PET/MRI (PET fused with gadolinium-enhanced Dixon): probable recurrence with high FDG uptake (arrows, SUVmean = 5.357; SUVmax = 6.979). Radiation-induced arteriopathy (FDG uptake, arrowheads). As MRI, DWI and PET evaluations were discordant, the case was considered as probably negative for recurrence. Surgical biopsies revealed granulation tissue in the neopharynx and fibrosis around the fistula. (**D**) Histological section (HE, original magnification 200×, left image): fibrosis with rare fibroblasts. Polarised light (right image, original magnification 100×): thick birefringent collagen bundles. Follow-up of 36 months further confirmed absence of recurrence

Thirty-two of the 43 recurrent HNSCCs (32/43,74%) had no obvious mucosal abnormality at endoscopy and PET/DWIMRI was essential in guiding the surgeon to select the most appropriate biopsy site. Correlation with whole-organ pathology revealed that true positive PET/DWIMRI assessments corresponded histologically to tumours located mainly beneath intact mucosa and with multicentric foci dispersed over large anatomical areas (Figs. 4 and 5). In 9/43 (20.9%) tumours, histology revealed microscopic perineural spread and intravascular tumour thrombi (Fig. 5). False-negative assessments (Table 2) were caused by oropharyngeal pTis (n = 1), laryngeal pT1 (n = 1) and oral cavity pT2 (n = 1) tumours; the mean diameter of missed tumours was 11 ± 9 mm. In retrospect, the readers were able to identify one of the three missed tumours. During the prospective readings, nine false-positive FDG-PET assessments were avoided due to absent restriction on DWI (n = 3) (Fig. 6) or due to absence of suspicious features on both DWI and MRI (n = 6). Likewise, two false-positive DWI assessments were avoided due to absent focal FDG uptake, whereas eight false-positive MRI interpretations were avoided due to non-suspicious PET and ADC (n = 6) or due to absent focal uptake only (n = 1). In three patients, however, combined PET/DWIMRI yielded four false-positive assessments (Table 2), which were caused by granulation tissue/ulcer (n = 2), infection/abscess (n = 1) or lymphoid hyperplasia (n = 1) mimicking recurrence on all three modalities. In retrospect, these four false-positive assessments could not have been avoided.

### Concordance analysis of MRI, DWI and PET readings

Morphological MRI, DWI and PET interpretations were considered concordant if the results on all three modalities (MRI, DWI *and* PET) were positive (rating ≥3) *or* if all results were negative (rating < 3). Results were considered discordant if two modalities were positive, one was negative or if one modality was positive and two were negative. Kappa coefficient for the concordance between the three methods was 0.71, indicating substantial agreement according to Landis and Koch [36].

Eighty-two of 105 (78.1%) lesions had concordant ratings. Forty of 105 (38.1%) lesions had concordant positive ratings, of which 39 (97.5%) corresponded to malignant tumours. Forty-two of 105 (40.0%) lesions had concordant negative ratings, of which 41 (97.6%) corresponded to benign lesions. Twenty-three of 105 (21.9%) lesions had discordant ratings, of which 20 (86.9%) corresponded to benign lesions. Among the discordant lesions, 14 were negative on two modalities and positive on one modality; 12 of these 14 lesions (85.7%) had a negative gold standard. Nine discordant lesions had two positive results and one negative result; eight of these nine lesions (88.9%) had a negative gold standard. Results were, therefore, more frequently concordant in recurrent tumours than in benign radiation-induced lesions (*p* = 0.0018).

### Imaging-based T-classification versus pT-classification and follow-up

The T-staging accuracy (95% CI) with combined PET/DWIMRI was 90.5% (83.2–95.3) (Table 3). Understaging occurred in 3/105 (2.9%), whereas overstaging occurred in 7/105 (6.6%) lesions, respectively. Kappa coefficient for the concordance between PET/DWIMRI and the final T-stage was 0.84 (*p* < 0.0001), indicating excellent agreement.

**Table 3:** T-classification accuracy of combined positron emission tomography (PET)/diffusion-weighted imaging (DWI) MRI (N = 105 lesions)

		T-classification pathology/follow-up**						
		T0	pT1*	pT2	pT3	pT4a	pT4b	Total
T-classification PET/DWIMRI	T0	58	2	1	0	0	0	61
	T1*	3	7	0	0	0	0	10
	T2	1	1	2	0	0	0	4
	T3	0	0	0	6	0	0	6
	T4a	0	0	0	2	21	0	23
	T4b	0	0	0	0	0	1	1
	Total	62	10	3	8	21	1	105
		Cohen’s kappa = 0.84, *p* < 0.0001						

* The only pTis lesion in this series was counted together with the T1 lesions

** Histology was the standard of reference in all 43 HNSCCs (salvage surgery in 37 tumours and excisional or diagnostic biopsy in six tumours). In 23 benign lesions, histology was the standard of reference (pT0), whereas for the remaining 39 benign lesions without histological proof, a negative follow-up (mean ± SD = 34 ± 8 months) was considered as indicating T0

## Discussion

In this prospective study, PET/DWIMRI had an excellent overall diagnostic performance and enabled accurate T-classification of recurrent tumours, therefore facilitating salvage surgery. Locally recurrent tumours had significantly lower ADCmean/ADCmin and significantly higher SUVmean/SUVmax than benign post-treatment lesions/complications, our results being in agreement with the literature [1, 5–7, 27, 31]. As tumour differentiation influences ADC values (poorly differentiated HNSCCs have lower ADCs), the low prevalence of well-differentiated tumours in this series may limit the overall validity of ADC measurements. Nevertheless, our ADCmean values were similar to those reported by others at 1.5 T and 3 T MRI, respectively [1, 5–7, 31]. Although AUCs for ADCmean and SUVmean/SUVmax were similar, there was no statistically significant correlation between ADC and SUV, therefore, our results further support evidence that these biomarkers are independent parameters in HNSCC [31, 35, 37]. Multivariate logistic regression analysis also showed that each binarised criterion (ADCmean ≤1.208, SUVmean ≥3.361) significantly improved tumour detection when added to the other criterion, thus additionally supporting the concept of ADC/SUV complementarity. The combination of SUV and ADC has also been recently used to stratify patients into risk groups, high SUVmax combined with high ADCmin being associated with worst prognosis [38].

We found no significant difference between visual assessment and quantitative assessment with ADC/SUV thresholds, although our thresholds were very similar to published thresholds based on DWIMRI and PET/CT. This finding may be of interest for clinical routine because the issue of SUV quantification using PET/MRI is not yet solved, recent publications having reported underestimation of SUVs with PET/MRI versus PET/CT [10, 11, 15, 16, 20, 26]. Moreover, SUV measurements may also be influenced by biological factors (blood glucose level, body size, breathing) and technological characteristics (scanner model, reconstruction parameters, dose calibration) [39]. Visual analysis of focal FDG uptake without threshold may thus be sufficient for the diagnosis of local recurrence in clinical routine. Visual assessment of FDG uptake without semi-quantitative measurements/thresholds is also used in many institutions for PET/CT scan interpretation [8, 29, 30, 40–42].

Meta-analyses evaluating PET and PET/CT for the follow-up of HNSCC found that the pooled sensitivity and NPV of PET and PET/CT for detecting residual/recurrent HNSCC at the primary site were very high, whereas the PPV was only in the range of 58.6–75% [29, 40–42]. In contrast to the PET/CT literature, data on the capability of DWIMRI to detect post-radio(chemo)therapy HNSCC recurrence are very sparse [5–7, 43]. Nevertheless, it was suggested that DWIMRI has high sensitivity/specificity, but variable PPV/NPV [5–7, 43]. A recent study by Queiroz et al. including patients with primary/recurrent HNSCC and other histological types reported that adding DWI information to PET/MRI may diminish specificity and overall diagnostic accuracy; the authors therefore concluded that DWI did not improve the diagnostic performance of PET/MRI [44]. We cannot confirm this observation. On the contrary, our excellent PET/DWIMRI performance for all analysed parameters (Table 2) including AUCs (Fig. 2) corroborates the fact that DWIMRI and PET provide complementary information in symptomatic irradiated HNSCC patients. DWI helped to avoid false-positive findings caused by FDG-uptake and nonspecific MRI morphology. It is well known that FDG-PET can lead to false-positive evaluations after radiotherapy as inflammatory cells contribute substantially to FDG-uptake [1, 9, 29, 30]. Possible explanations regarding the discrepancy between our results and the results of Queiroz et al [44] include: different histology, post-treatment imaging only versus pre- and post-treatment imaging, evaluation of local recurrence only versus evaluation of tumours, lymph nodes and metastases, different DWI parameters and use of different diagnostic criteria (multiparametric complementarity in this study versus ‘MRI, PET *or* DWI positivity’). While a combination of criteria using the ‘*or*’-conjunction may increase sensitivity, it invariably yields lower specificity, as shown by our analysis of ADCmean/SUVmean thresholds.

Prospective interpretation of multiparametric data is challenging, in particular if morphological MRI, DWI and PET findings are discrepant (21.9% in this series). Currently there is no consensus on how to deal with such discrepant information, whether it should be preferred to rely on morphological MRI, DWI or PET. This diagnostic uncertainty can lead to unnecessary biopsy in irradiated tissues with the risk of precipitating infection. Our study may show a way to manage concordant/discordant readings as positive concordant results with all techniques added diagnostic certainty and corresponded to recurrent tumours whereas negative concordant results and discordant results most often corresponded to benign lesions. This approach could also be applied in indeterminate/suspicious FDG-PET/CT readings, in which case a high ADC revealed by DWIMRI would instead lead to a wait and see policy instead of biopsy. Larger patient series are, however, necessary to substantiate this approach.

Recurrent HNSCCs tend to occur submucosally with multicentric tumour foci dispersed over large anatomical areas, a growth pattern that is different from the rather concentrical growth of primary HNSCCs [45]. Our study confirms this reported growth pattern and suggests that this distinct histological feature accounts for the ill-defined tumour aspect at imaging. In our study, PET/DWIMRI had a high staging accuracy with excellent agreement between imaging-based and pT stage. This is in contrast to previous reports suggesting that MRI or CT may grossly underestimate local recurrence leading to inadequate surgery in up to 50% of patients [46]. Possible explanations for the high diagnostic PET/DWIMRI performance and high T-staging accuracy include the routine use of high-resolution images, clearly defined diagnostic criteria with precise analysis of signal intensity and enhancement patterns, increased diagnostic confidence due to multiparametric information and evaluation by experienced readers. The fact that only experienced readers interpreted the images may constitute a limitation of this study, and it may be necessary to perform a multi-observer study to evaluate whether our results are also reproducible by readers who are less familiar with HN imaging.

The purpose of this study was to evaluate the diagnosis of local HNSCC recurrence. Detection of nodal recurrence requires detailed systematic correlation with neck dissection specimen on a level-by-level and node-by-node basis. Although this is also important from a clinical point of view, the related questions are beyond the scope of this report and require a separate analysis.

In summary, PET/DWIMRI has an excellent diagnostic performance for the detection of HNSCC recurrence after radio(chemo)therapy with excellent agreement between imaging-based and pathological T-stage provided appropriate diagnostic criteria are applied. Results of our study show that positive concordant results with MRI, DWI and PET correspond to locally-recurrent HNSCC, negative concordant results correspond to absent recurrence, and discordant results rather correspond to benign post-radio(chemo)therapy lesions/complications.

## References

[1] Varoquaux A, Rager O, Dulguerov P (2015). Diffusion-weighted and PET/MR imaging after radiation therapy for malignant head and neck tumors. Radiographics.

[2] Omura G, Saito Y, Ando M (2014). Salvage surgery for local residual or recurrent pharyngeal cancer after radiotherapy or chemoradiotherapy. Laryngoscope.

[3] Camisasca DR, Silami MA, Honorato J, Dias FL, de Faria PA, Lourenco Sde Q (2011). Oral squamous cell carcinoma: clinicopathological features in patients with and without recurrence. ORL J Otorhinolaryngol Relat Spec.

[4] Guo T, Qualliotine JR, Ha PK (2015). Surgical salvage improves overall survival for patients with HPV-positive and HPV-negative recurrent locoregional and distant metastatic oropharyngeal cancer. Cancer.

[5] Tshering Vogel DW, Zbaeren P, Geretschlaeger A, Vermathen P, De Keyzer F, Thoeny HC (2013). Diffusion-weighted MR imaging including bi-exponential fitting for the detection of recurrent or residual tumour after (chemo)radiotherapy for laryngeal and hypopharyngeal cancers. Eur Radiol.

[6] Abdel Razek AA, Kandeel AY, Soliman N (2007). Role of diffusion-weighted echo-planar MR imaging in differentiation of residual or recurrent head and neck tumors and posttreatment changes. AJNR Am J Neuroradiol.

[7] Vandecaveye V, De Keyzer F, Nuyts S (2007). Detection of head and neck squamous cell carcinoma with diffusion weighted MRI after (chemo)radiotherapy: correlation between radiologic and histopathologic findings. Int J Radiat Oncol Biol Phys.

[8] Abgral R, Querellou S, Potard G (2009). Does 18F-FDG PET/CT improve the detection of posttreatment recurrence of head and neck squamous cell carcinoma in patients negative for disease on clinical follow-up?. J Nucl Med.

[9] Purohit BS, Ailianou A, Dulguerov N, Becker CD, Ratib O, Becker M (2014). FDG-PET/CT pitfalls in oncological head and neck imaging. Insights Imaging.

[10] Zaidi H, Becker M (2016). The promise of hybrid PET/MRI: technical advances and clinical applications. IEEE Signal Proc Mag.

[11] Becker M, Zaidi H (2014). Imaging in head and neck squamous cell carcinoma: the potential role of PET/MRI. Br J Radiol.

[12] Vargas MI, Becker M, Garibotto V (2013). Approaches for the optimization of MR protocols in clinical hybrid PET/MRI studies. MAGMA.

[13] Covello M, Cavaliere C, Aiello M (2015). Simultaneous PET/MR head-neck cancer imaging: preliminary clinical experience and multiparametric evaluation. Eur J Radiol.

[14] Purohit BS, Dulguerov P, Burkhardt K, Becker M (2014). Dedifferentiated laryngeal chondrosarcoma: combined morphologic and functional imaging with positron-emission tomography/magnetic resonance imaging. Laryngoscope.

[15] Bailey DL, Antoch G, Bartenstein P (2015). Combined PET/MR: the real work has just started. summary report of the third international workshop on PET/MR imaging; February 17-21, 2014, Tubingen, Germany. Mol Imaging Biol.

[16] Varoquaux A, Rager O, Poncet A (2014). Detection and quantification of focal uptake in head and neck tumours: (18)F-FDG PET/MR versus PET/CT. Eur J Nucl Med Mol Imaging.

[17] Queiroz MA, Hullner M, Kuhn F (2014). PET/MRI and PET/CT in follow-up of head and neck cancer patients. Eur J Nucl Med Mol Imaging.

[18] Drzezga A, Souvatzoglou M, Eiber M (2012). First clinical experience with integrated whole-body PET/MR: comparison to PET/CT in patients with oncologic diagnoses. J Nucl Med: Off Publ, Soc Nucl Med.

[19] Kubiessa K, Purz S, Gawlitza M (2014). Initial clinical results of simultaneous 18F-FDG PET/MRI in comparison to 18F-FDG PET/CT in patients with head and neck cancer. Eur J Nucl Med Mol Imaging.

[20] Weber WA (2014). PET/MR imaging: a critical appraisal. J Nucl Med.

[21] Vandecaveye V, Dirix P, De Keyzer F (2012). Diffusion-weighted magnetic resonance imaging early after chemoradiotherapy to monitor treatment response in head-and-neck squamous cell carcinoma. Int J Radiat Oncol Biol Phys.

[22] Vandecaveye V, De Keyzer F, Vander Poorten V (2009). Head and neck squamous cell carcinoma: value of diffusion-weighted MR imaging for nodal staging. Radiology.

[23] Lambrecht M, Van Calster B, Vandecaveye V (2012). Integrating pretreatment diffusion weighted MRI into a multivariable prognostic model for head and neck squamous cell carcinoma. Radiother Oncol.

[24] Maehara M, Ikeda K, Kurokawa H (2014). Diffusion-weighted echo-planar imaging of the head and neck using 3-T MRI: investigation into the usefulness of liquid perfluorocarbon pads and choice of optimal fat suppression method. Magn Reson Imaging.

[25] Brandão S, Nogueira L, Matos E (2015). Fat suppression techniques (STIR vs SPAIR) on diffusion weighted imaging of breast lesions at 3.0 T: preliminary experience. Radiol Med.

[26] Arabi H, Rager O, Alem A, Varoquaux A, Becker M, Zaidi H (2015). Clinical assessment of MR-guided 3-class and 4-class attenuation correction in PET/MR. Mol Imaging Biol.

[27] Thoeny HC, De Keyzer F, King AD (2012). Diffusion-weighted MR imaging in the head and neck. Radiology.

[28] Becker M, Zbären P, Casselman JW, Kohler R, Dulguerov P, Becker CD (2008). Neoplastic invasion of laryngeal cartilage: reassessment of criteria for diagnosis at MR imaging. Radiology.

[29] Sheikhbahaei S, Taghipour M, Ahmad R (2015). Diagnostic accuracy of follow-up FDG PET or PET/CT in patients with head and neck cancer after definitive treatment: a systematic review and meta-analysis. AJR Am J Roentgenol.

[30] Robin P, Abgral R, Valette G (2015). Diagnostic performance of FDG PET/CT to detect subclinical HNSCC recurrence 6 months after the end of treatment. Eur J Nucl Med Mol Imaging.

[31] Varoquaux A, Rager O, Lovblad KO (2013). Functional imaging of head and neck squamous cell carcinoma with diffusion-weighted MRI and FDG PET/CT: quantitative analysis of ADC and SUV. Eur J Nucl Med Mol Imaging.

[32] Brierley JD, Gospodarowicz MK, Wittekind C (2016). TNM Classification of Malignant Tumours 8th ed. Wiley-Blackwell, Hoboken

[33] Zhou X-H, Obuchowksi NA, McClish DK (2011). Statistical Methods in Diagnostic Medicine.

[34] Obuchowski NA (1997). Nonparametric analysis of clustered ROC curve data. Biometrics.

[35] Perkins NJ, Schisterman EF (2006). The inconsistency of "optimal" cutpoints obtained using two criteria based on the receiver operating characteristic curve. Am J Epidemiol.

[36] Landis JR, Koch GG (1977). The measurement of observer agreement for categorical data. Biometrics.

[37] Fruehwald-Pallamar J, Czerny C, Mayerhoefer ME (2011). Functional imaging in head and neck squamous cell carcinoma: correlation of PET/CT and diffusion-weighted imaging at 3 Tesla. Eur J Nucl Med Mol Imaging.

[38] Preda L, Conte G, Bonello L (2016). Combining standardized uptake value of FDG-PET and apparent diffusion coefficient of DW-MRI improves risk stratification in head and neck squamous cell carcinoma. Eur Radiol.

[39] Adams MC, Turkington TG, Wilson JM (2010). A systematic review of the factors affecting accuracy of SUV measurements. AJR Am J Roentgenol.

[40] Cheung PK, Chin RY, Eslick GD (2016). Detecting residual/recurrent head neck squamous cell carcinomas using PET or PET/CT: systematic review and meta-analysis. Otolaryngol Head Neck Surg.

[41] Isles MG, McConkey C, Mehanna HM (2008). A systematic review and meta-analysis of the role of positron emission tomography in the follow up of head and neck squamous cell carcinoma following radiotherapy or chemoradiotherapy. Clin Otolaryngol.

[42] Gupta T, Master Z, Kannan S (2011). Diagnostic performance of post-treatment FDG PET or FDG PET/CT imaging in head and neck cancer: a systematic review and meta-analysis. Eur J Nucl Med Mol Imaging.

[43] Driessen JP, van Kempen PM, van der Heijden GJ (2015). Diffusion-weighted imaging in head and neck squamous cell carcinomas: a systematic review. Head Neck.

[44] Queiroz MA, Hüllner M, Kuhn F (2014). Use of diffusion-weighted imaging (DWI) in PET/MRI for head and neck cancer evaluation. Eur J Nucl Med Mol Imaging.

[45] Zbaren P, Nuyens M, Curschmann J, Stauffer E (2007). Histologic characteristics and tumor spread of recurrent glottic carcinoma: analysis on whole-organ sections and comparison with tumor spread of primary glottic carcinomas. Head Neck.

[46] Zbaren P, Christe A, Caversaccio MD, Stauffer E, Thoeny HC (2007). Pretherapeutic staging of recurrent laryngeal carcinoma: clinical findings and imaging studies compared with histopathology. Otolaryngol Head Neck Surg.

